# High Throughput Sequencing of Small RNAs in the Two *Cucurbita* Germplasm with Different Sodium Accumulation Patterns Identifies Novel MicroRNAs Involved in Salt Stress Response

**DOI:** 10.1371/journal.pone.0127412

**Published:** 2015-05-26

**Authors:** Junjun Xie, Bo Lei, Mengliang Niu, Yuan Huang, Qiusheng Kong, Zhilong Bie

**Affiliations:** College of Horticulture and Forestry, Huazhong Agricultural University/Key Laboratory of Horticultural Plant Biology, Ministry of Education, Wuhan, 430070, P. R. China; East Carolina University, UNITED STATES

## Abstract

MicroRNAs (miRNAs), a class of small non-coding RNAs, recognize their mRNA targets based on perfect sequence complementarity. MiRNAs lead to broader changes in gene expression after plants are exposed to stress. High-throughput sequencing is an effective method to identify and profile small RNA populations in non-model plants under salt stresses, significantly improving our knowledge regarding miRNA functions in salt tolerance. Cucurbits are sensitive to soil salinity, and the *Cucurbita* genus is used as the rootstock of other cucurbits to enhance salt tolerance. Several cucurbit crops have been used for miRNA sequencing but salt stress-related miRNAs in cucurbit species have not been reported. In this study, we subjected two *Cucurbita* germplasm, namely, N12 (*Cucurbita*. *maxima* Duch.) and N15 (*Cucurbita*. *moschata* Duch.), with different sodium accumulation patterns, to Illumina sequencing to determine small RNA populations in root tissues after 4 h of salt treatment and control. A total of 21,548,326 and 19,394,108 reads were generated from the control and salt-treated N12 root tissues, respectively. By contrast, 19,108,240 and 20,546,052 reads were obtained from the control and salt-treated N15 root tissues, respectively. Fifty-eight conserved miRNA families and 33 novel miRNAs were identified in the two *Cucurbita* germplasm. Seven miRNAs (six conserved miRNAs and one novel miRNAs) were up-regulated in salt-treated N12 and N15 samples. Most target genes of differentially expressed novel miRNAs were transcription factors and salt stress-responsive proteins, including dehydration-induced protein, cation/H^+^ antiporter 18, and CBL-interacting serine/threonine-protein kinase. The differential expression of miRNAs between the two *Cucurbita* germplasm under salt stress conditions and their target genes demonstrated that novel miRNAs play an important role in the response of the two *Cucurbita* germplasm to salt stress. The present study initially explored small RNAs in the response of pumpkin to salt stress, and provided valuable information on novel miRNAs and their target genes in *Cucurbita*.

## Introduction

MicroRNAs (miRNAs), a class of small non-coding RNAs widely distributed throughout the plant kingdom and highly evolutionarily conserved, recognize their mRNA targets based on perfect sequence complementarity. MiRNAs cause either transcriptional or post-transcriptional gene silencing [1, 2]. Since the first plant miRNAs were reported in *Arabidopsis thaliana*, the identified miRNAs in various plant species have sharply increased. To date, a total of 13,618 plant miRNAs from 72 species have been deposited in miRBase (miRBase Release20.0, http://www.mirbase.org/). MiRNA-mediated gene silencing is a conserved regulatory mechanism underlying plant responses to biotic and abiotic stresses. Meanwhile, most target genes of miRNAs are transcription factors and receptor proteins; thus, miRNAs function as early signaling components that can ultimately lead to extensive changes in gene expression after plants are exposed to stress [1, 3–5].

Salinity, a major abiotic stress, affects more than 80 million hectare of arable lands worldwide. Soil salinity severely reduces the yield and productivity of many crops, resulting in an annual global loss of US$11,000 million in 2011 [6–8]. As sessile organism, plants have evolved multiple regulatory profiles to resist salt stress and sustain their growth. Different genes, biomolecules, and compounds that can be altered to make plants resistant to salt have been identified [9]. Some specific miRNAs are also over- or under-expressed in plant to cope with stress [10]. Several salinity stress-regulated miRNAs have been identified in model plants. In Arabidopsis, miR156, miR171, and miR319 are up-regulated in response to salt stress, and the accumulation of miR398 decreases [11]. In maize, the members of the MiR156, MiR164, and MiR396 families are down regulated, whereas the MiR168 family is up regulated in salt-shocked maize roots [12]. In rice, miR169, which may be regulated by abscisic acid (ABA), is up-regulated under high salinity stress, and participates in the transcriptional regulation of a large number of genes through selective cleaving of a CCAAT-box binding transcription factor [13]. Previous studies indicated that miRNAs may regulate protein-coding genes and utilize gene networks to respond to salt stresses [2]. Meanwhile, high-throughput next-generation sequencing which can identify most miRNAs and easily predict species-specific miRNAs has been used in soybean, cotton, sugarcane and switchgrass to systematically identify the responsiveness of miRNAs to salt stresses, thereby significantly improving our knowledge regarding miRNA functions in salt tolerance [14–17].

Cucurbits are popular and economically important fruit vegetables that are widely cultivated in almost all regions with arable lands [18]. Cucurbits are glycophytes; therefore, most cucurbits (particularly watermelon, melon, and cucumber) are sensitive to soil salinity. The *Cucurbita* genus is used as the rootstock of other cucurbits by accumulating Na^+^ in the rootstock, and reducing Na^+^ concentrations in shoots to enhance the salt tolerance [19–21]. Several studies have reported miRNAs in cucurbit crops (including cucumber, melon, watermelon, and pumpkin) [22–27]. However, abiotic stresses particularly salt stress involved miRNAs in cucurbit species have not been reported yet. In the present study, two *Cucurbita* germplasm, namely, N12 (*Cucurbita*. *maxima* Duch.) and N15 (*Cucurbita*. *moschata* Duch.), were used to detect differentially expressed miRNAs in root tissues during the early salt respond period. These two varieties exhibited different Na^+^ accumulation patterns. N12 accumulated Na^+^ in the shoots, whereas N15 accumulated Na^+^ in the roots. This study provides useful information to further investigate *Cucurbita* miRNAs, thereby elucidating their possible regulatory roles under salt stress conditions. The results obtained can be utilized in rootstocks improvement and breeding programs.

## Materials and Methods

### Plant materials and salt stress treatment

Two *Cucurbita* germplasm (Inbred lines constructed by our group), namely, N12 (*C*. *maxima* Duch.) and N15 (*C*. *moschata* Duch.), were used in this experiment. The plant material was cultivated following previously described methods with slight modification [28, 29]. In brief, the seeds were soaked in water for 4 h and then incubated in the dark at 30°C until germination. The germinated seeds were directly sown and grown on sponge dices of 2.5 cm × 2.5 cm × 1.5 cm (length × width × height) instead of on a substrate to avoid potential NaCl contamination before salt-stress treatment. Deionized water was supplemented until the root grew out of the sponge. The seedlings were then transferred to 8 L plastic containers (nine plants per container) containing full-strength Hoagland solution in a climatic chamber with a 16 h light/8 h dark photoperiod at 28°C /20°C day/night temperatures and relative humidity of 75% [30]. The nutrient solutions were replenished every 5 d and continuously aerated with an air pump. At the three true-leaf stage, the Na^+^ concentration was increased to 100mM by gradually adding NaCl to the full-strength Hoagland solution. To prevent the potential effects of diurnal rhythm on hydraulic properties, we used the seedlings grown on full-strength Hoagland solution as control at the same sampling points of the treatments. The samples were collected from six seedlings to obtain three biological replicates.

### Measurement of Na^+^ content in different tissues

Seedlings of N12 and N15were randomly harvested to determine the Na^+^ content in the roots, stems, and leaves after 10d of salt-stress treatment in each biological replicate. For each plant, the roots, stems, and leaves were cut and dried at 75°C until a constant-weight was obtained. Each sample was digested in 10 ml 98% H_2_SO_4_ and 3 ml 30% H_2_O_2_ for 5 h [31]. After filtration, the samples were diluted with deionized water to obtain a proper gradient for measurement. The Na^+^ content was determined using an atomic absorption spectrophotometer (Varian spectra AA 220; Varian, USA). ANOVA was performed using SAS software (SAS Institute, USA). Duncan’s multiple range test (p<0.05) was used to established differences between the treatment means.

### RNA preparation for sequencing

Control and 4 h salt-stress treatment seedlings of N12 and N15 were randomly harvested to extract total RNA for sequencing in each biological replicate. Total RNA was isolated using TRIzol reagent (Invitrogen, USA) following the manufacturer’s instructions. Total RNA was isolated from N12 and N15 roots under salt stress or control. Each biological replicate was equally mixed and designated as 24hNR, 24hR, 54hNR, and 54hR (24hNR, N12 root samples of salt stress; 24hR, N12 root samples of control; 54hNR, N15 root samples of salt stress; and 54hR, N15 root samples of control). For each sample, a minimum of 40 mg of total RNA was transported to the Beijing Genomics Institute (BGI, Shenzhen, China) for Illumina sequencing (commercial service).

### Identification of conserved and novel miRNAs

Four small RNA libraries (24hNR, 24hR, 54hNR, and 54hR) were sequenced using Illumina sequencing technology at the BGI. After removing the low-quality reads and adapter sequences from the Illumina sequencing reads, unique sequences of 18–30 nt were used for further analysis.

First, unique sequences were mapped to the transcriptome sequences of *C*. maxima Duch. and *C*. moschata Duch. with less than 2 bp mismatch using SOAP [32]. Then the unique sequences were also queried against ribosomal and transfer RNAs from NCBI GenBank (GenBank: http://www.ncbi.nlm.nih.gov/blast/Blast.cgi), whereas the rRNA, tRNA, snRNA, and snoRNA sequences were obtained from Rfam (Rfam: www.sanger.ac.uk/software/Rfam). Small RNA sequences that exactly matched these sequences were removed from the analysis. The remaining unique small RNA sequences were analysed to identify conserved miRNAs by BLAST against the conserved plant miRNAs in the miRBase database (release 18.0, November 2011). MIREAP software (https://sourceforge.net/projects/mireap/) was used to predict novel miRNAs. The MIREAP parameters were set as follows: (1) a characteristic stem-loop structure was formed; (2) the length of the miRNA sequence was 20–23 nt; (3) the maximal free energy allowed for the miRNA precursor was -20 kcal mol^-1^; (4) the minimum number of common base pairs between miRNA and miRNA* was 16, with no more than four bulges; and (5) the maximum asymmetry of the miRNA/miRNA* duplex was four bases.

### Differential expression of miRNAs between the two *Cucurbita* germplasm under salt stress

The miRNA expression levels between two samples were compared, and the procedures are as follows [33]: (1) The expression of miRNA in the two samples was normalized [CK (24hR or 54hR) and treat (24hNR or 54hNR)] to obtain the expression of transcript per million (TPM) using the following formula: Normalized expression = actual miRNA count/total count of clean reads×1,000,000; (2) The fold changes from the normalized expression was calculated using the following formula: Fold change = log2 (treatment/control). The fold change was modified to 0.01 if the normalized expression was zero; however, the samples were not subjected to differential analysis if the expression of some miRNAs was less than 1.

### Prediction of target genes of differentially expressed miRNAs and gene ontology (GO) enrichment

Transcriptome sequences of *C*. *maxima* Duch. and *C*. *moschata* Duch. were used to identify the target genes of miRNA. The psRNA Target program (http://plantgrn.noble.org/psRNATarget) was used to predict target genes of miRNAs according to previously established rules [34, 35]. Sequencing data have been submitted into the NCBI/SRA database with accession numbers SRP048781 (small RNA sequences) and SRP049398 (transcriptome sequences).

GO enrichment analysis was used on the predicted target genes and proved that all GO terms were significantly enriched and genes corresponded to certain biological functions.

### qRT-PCR validation of miRNAs and their target mRNAs

Total RNA was extracted from root tissue of the control and 4 h salt-stress treatment seedlings of N12 and N15 for RT-PCR in each biological replicate. Poly (A)-tailed qRT-PCR is a frequently used real-time PCR method for quantifying the expression of plant miRNAs [36]. We selected a miRNA First-Strand cDNA Synthesis SuperMix (Trans, China) which supports the addition of poly (A) tails for reverse transcription of miRNAs. Reverse transcription of miRNA was completed following the manufacturer’s instructions. The forward primers were designed based on the mature miRNA sequences (S1 Table), and the reverse primers were provided in the kit. U6RNA was used as an internal reference. Reverse transcription of mRNA was conducted using first strand cDNA synthesis of One-Step gDNA Removal and cDNA Synthesis SuperMix (Trans, China) following the manufacturer’s instructions. The primers for the target mRNAs are shown in S1 Table. EF1-α was used as an internal reference. The qRT-PCR reactions were performed on a LightCycler480 System (Roche), with Top Green qRCR SuperMix (Trans, China) in a 10 μL reaction volume containing 2 μL of diluted cDNA, 5 μL of 2×Top Green qRCR SuperMix and 1.5 μL of each primer (2 μM). The miRNA PCR reaction conditions comprised an initial denaturation at 94°C for 30 s, followed by 45 cycles of 94°C for 5 s and 60°C for 30 s. The mRNA PCR reaction conditions comprised an initial denaturation at 94°C for 30 s, followed by 40 cycles of 94°C for 5 s, 56°C for 30 s and 72°C for 10 s. The relative level of expression was calculated using the formula 2^-ΔΔct^ [37].

## Results

### N12 and N15 exhibited different Na^+^ accumulation patterns

**Fig 1:**
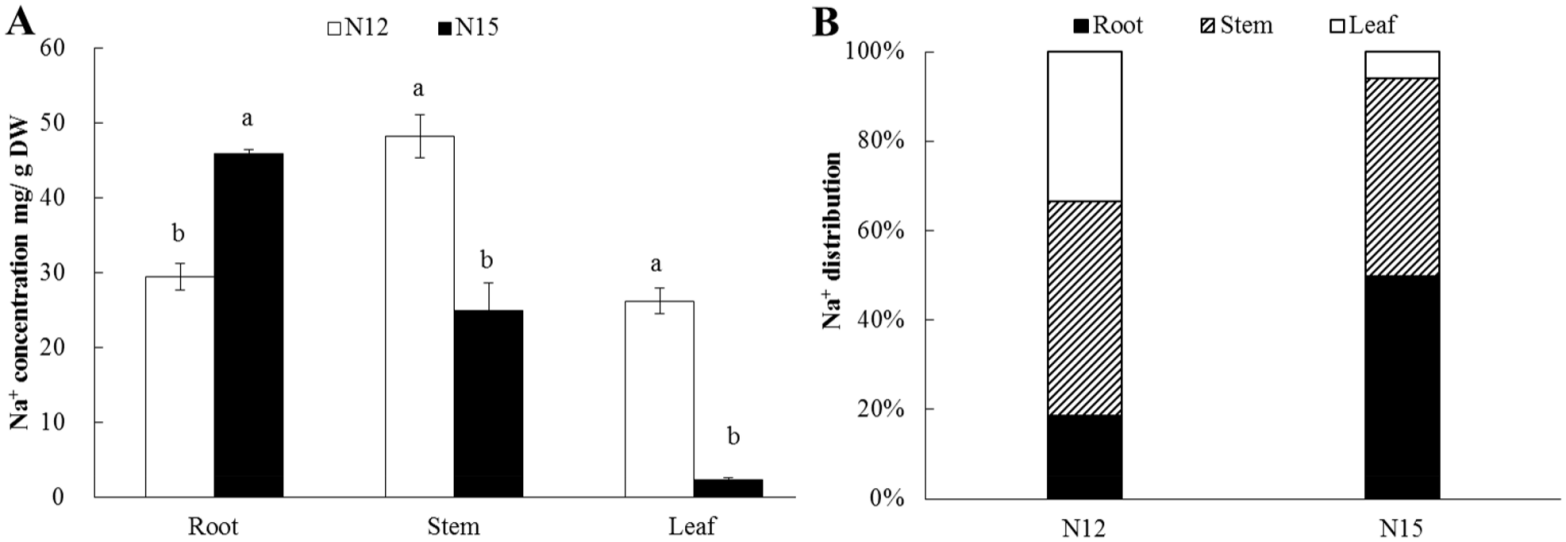
Na^+^ concentrations in the leaves, stems and roots (A) and Na^+^ distribution (B) in N12 and N15 treated with 100 mM NaCl for 10d. Data represent the mean ± SE (*n* = 6). Different letters indicate significant differences between treatments using Duncan’s multiple range test at p<0.05.

To characterize the differences between N12 and N15 in terms of salt-stress tolerance, the three true-leaf stage seedlings were treated with full-strength Hoagland solution containing 100mM NaCl for 10 days. Result showed that the concentration of Na^+^ in root of N12 was significantly higher than N15. However, the concentration of Na^+^ in stem and leaf of N12 were significantly lower than that N15 (Fig 1A). Na^+^ distribution in whole plant showed clear differences between the two *Cucurbita* germplasm (Fig 1B). Almost 80% of Na^+^ was accumulated in N12 shoots, whereas about 50% Na^+^ accumulated in N15 roots. These results demonstrated that N12 and N15 exhibited different Na^+^ accumulation patterns under salt stress conditions; N12 accumulated Na^+^ in the shoots, whereas N15 accumulated Na^+^ in the roots.

### Sequencing output and analysis of small RNAs

To identify miRNAs in N12 and N15 under salt stress conditions, we performed Illumina sequencing on the small RNA libraries after 4 h of treatment and control. A total of 21,548,326, 19,394,108, 19,108,240, and 20,546,052 reads were generated from the 24hR, 24hNR 54hR and 54hNR libraries, respectively (Table 1). The clean reads exhibited lengths of 15–30 nt, and represented more than 4,700,000 unique sequences in each library.

**Table 1:** Summary of data cleaning produced by small RNA sequencing in four libraries.

Type	24hR		24hNR		54hR		54hNR	
	count	percent	count	percent	count	percent	count	percent
total reads	21548326		19394108		19108240		20546052	
high quality	21451529	100%	19326099	100%	19040294	100%	20484590	100%
3'adapter null	8356	0.04%	5517	0.03%	5102	0.03%	4258	0.02%
insert null	5330	0.02%	5275	0.03%	4259	0.02%	2579	0.01%
5'adapter contaminants	57605	0.27%	71868	0.37%	79498	0.42%	57398	0.28%
smaller than 18nt	55442	0.26%	41472	0.21%	121846	0.64%	160245	0.78%
polyA	2225	0.01%	2068	0.01%	2191	0.01%	2010	0.01%
clean reads	21322571	99.40%	19199899	99.35%	18827398	98.88%	20258100	98.89%

**Fig 2:**
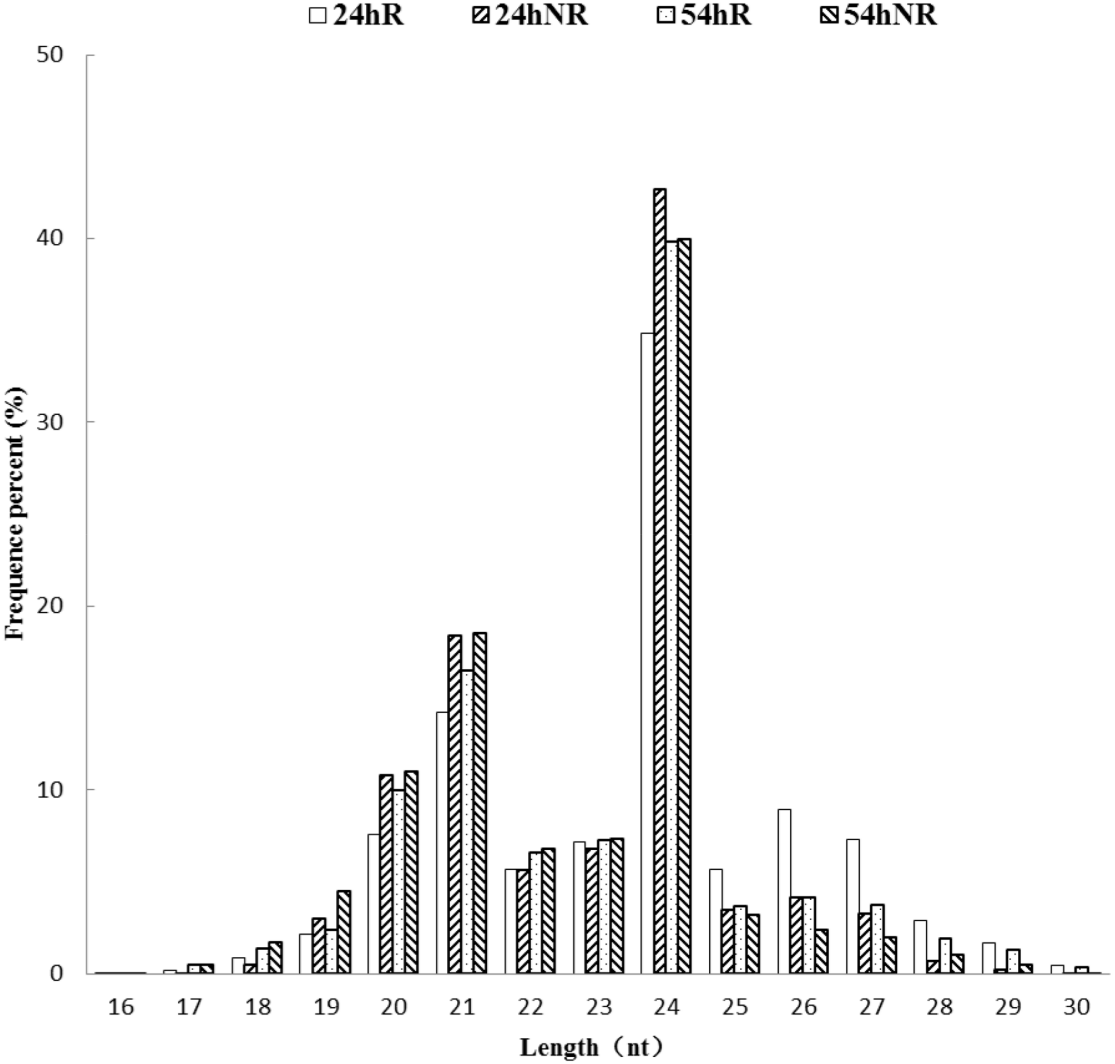
Length distribution of small RNAs in the four libraries. X-axis, length of sRNA distribution; Y-axis, percent frequency of raw reads. 24hR, N12 root under control library; 24hNR, N12 root under salt stress treatment library; 54hR, N15 root under control library; 54hNR, N15 root under salt stress treatment library.

The genome sequences of *Cucurbita* germplasm have not been released yet, so the transcriptome sequences of *C*. maxima Duch. and *C*. moschata Duch. were used as genome sequences for genome mapping. A total of 11,156,543 (52.32%), 8,633,219 (44.96%), 8,149,718 (43.29%) and 8,854,877 (43.71%) unique small RNA reads were mapped to the genome in the 24hR, 24hNR, 54hR, and 54hNR libraries, respectively. The numbers and proportions for rRNAs, tRNAs, snRNAs, and snoRNAs are shown in Table 2. All four libraries demonstrated similar distributions to other RNA families (Fig 2).

**Table 2:** Distribution of small RNAs among different categories in four libraries.

Category	24hR		24hNR		54hR		54hNR	
	Unique sRNAs	Total sRNAs	Unique sRNAs	Total sRNAs	Unique sRNAs	Total sRNAs	Unique sRNAs	Total sRNAs
Total	4729791(100%)	21322571(100%)	5199297(100%)	19199899(100%)	5129398(100%)	18827398(100%)	5763496(100%)	20258100(100%)
miRNA	1528(0.03%)	3474056(16.29%)	1645(0.03%)	4607307(24%)	1562(0.03%)	3447339(18.31%)	1820(0.03%)	3795697(18.74%)
rRNA	165284(3.49%)	3293825(15.45%)	115780(2.23%)	1357167(7.07%)	161859(3.16%)	2090916(11.11%)	178687(3.1%)	2572974(12.7%)
repeat	25(0%)	29(0%)	26(0%)	31(0%)	24(0%)	28(0%)	27(0%)	28(0%)
snRNA	4074(0.09%)	24545(0.12%)	3601(0.07%)	16465(0.09%)	4432(0.09%)	20128(0.11%)	6118(0.11%)	31339(0.15%)
snoRNA	897(0.02%)	3733(0.02%)	1040(0.02%)	4135(0.02%)	867(0.02%)	2505(0.01%)	1113(0.02%)	3763(0.02%)
tRNA	51800(1.1%)	971311(4.56%)	28495(0.55%)	630618(3.28%)	41621(0.81%)	724582(3.85%)	37214(0.65%)	831432(4.1%)
unann	4497849(95.1%)	13514057(63.38%)	5039531(96.93%)	12537910(65.3%)	4910061(95.72%)	12415164(65.94%)	5526881(95.89%)	12817580(63.27%)

### Identification of conserved miRNAs

**Fig 3:**
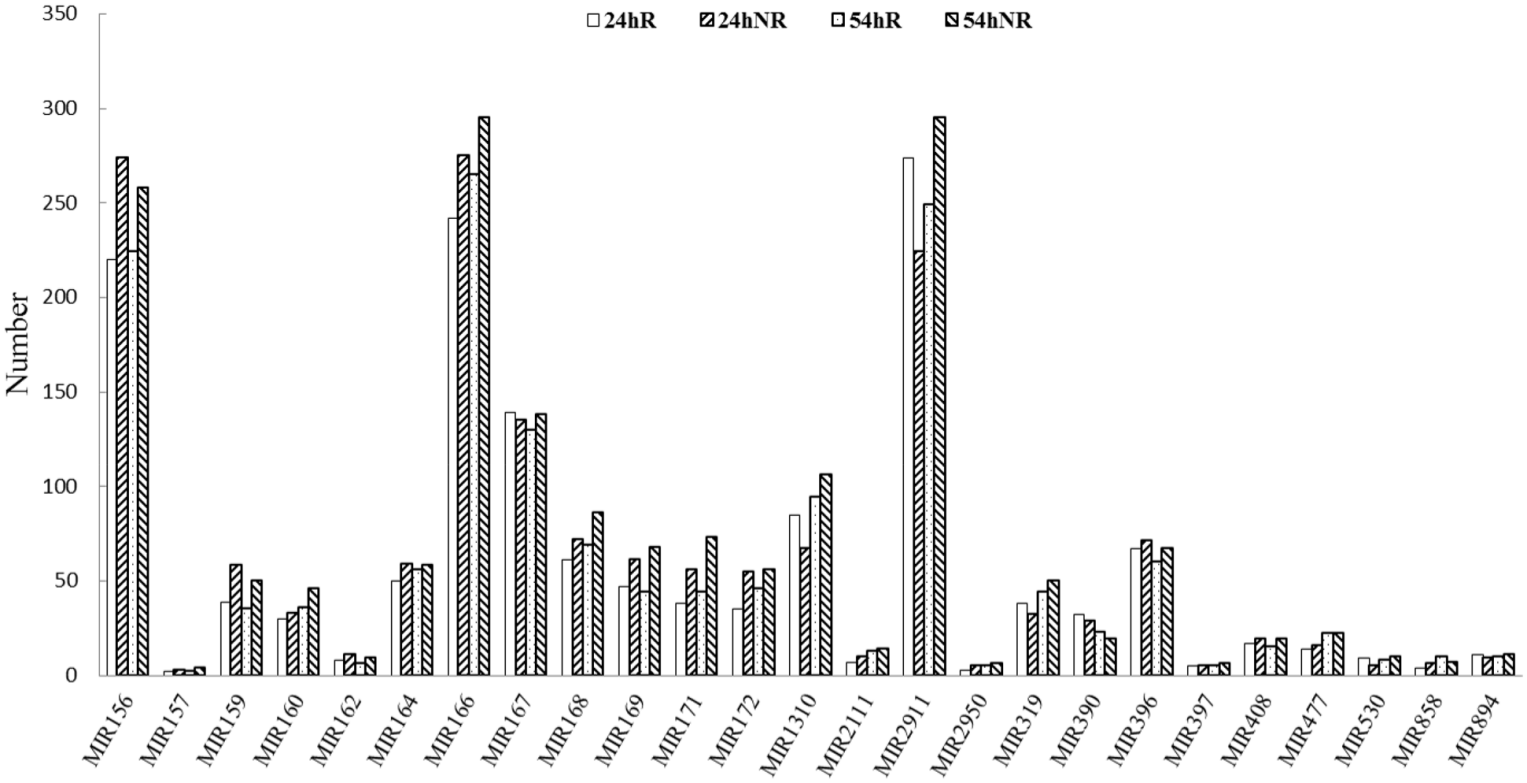
Numbers of identical miRNA members in each conserved miRNA family in the four libraries. 24hR, N12 root under control library; 24hNR, N12 root under salt stress treatment library; 54hR, N15 root under control library; 54hNR, N15 root under salt stress treatment library.

The unique sRNA sequences were compared with the conserved plant miRNAs to identify conserved miRNAs in the four libraries. A total of 1529, 1643, 1561, and 1820 unique miRNA sequences from 43, 47, 46, and 40 miRNA families were identified in 24hR, 24hNR, 54hR, and 54hNR, respectively (S2 Table). MiR156, MiR166, MiR167, MiR2911, and MiR396 were relatively abundant, whereas MiR157, MiR2950 and MiR397 were limited (Fig 3).

In the four libraries, miRNAs exhibited a broad range of read count. In these conserved miRNA families, MiR156, MiR166, MiR167, and MiR168 contained the most abundant component with more than 10,000 reads. In 24hR, miR156j contained about 100,000 reads, and it was also abundant with 140,000 reads in 24hNR and 54hNR. The miRNA families MiR159, MiR171, MiR390, MiR396, and MiR894 contained thousands of reads, whereas seven families (MiR162, MiR2111, MiR319, MiR397, MiR408, MiR477, and MiR858) comprised more than 100 reads (Table 3). Moreover, some miRNAs showed clear responses to salt treatments. Compared to control, miRNA157d, miRNA159a and miRNA160b contained twice as many reads in the two *Cucurbita* germplasm under salt treatment, and miRNA171c comprised quadruple reads. However, some miRNAs, such as miRNA162, miRNA166a, and miRNA167d, showed no response after salt treatment.

**Table 3:** Summary of conserved miRNAs in four libraries.

miRNA Family	Name	Length	miRNA sequence	24hR (reads)	24hNR (reads)	54hR (reads)	54hNR (reads)	Homology by specie
**miR1310**	miR1310	20	ACTTTAAATAGGTAGGACGG	446	134	156	226	Helianthus annuus
**miR156**	miR156b	21	TTGACAGAAGATAGAGAGCAC	492183	605686	210360	269073	Cucumis melo
	miR156f	21	CTGACAGAAGATAGAGAGCAC	146566	285017	151452	238385	Cucumis melo
	miR156j	20	TGACAGAAGAGAGTGAGCAC	976186	1467160	1341542	1453029	Cucumis melo
**miR157**	miR157d	22	CTGACAGAAGATAGAGAGCACT	810	2241	498	918	Arabidopsis lyrata
**miR159**	miR159a	21	TTTGGATTGAAGGGAGCTCTA	977	1151	1408	2079	Cucumis melo
**miR160**	miR160b	21	GCGTATGAGGAGCCAAGCATA	423	2196	482	1856	Cucumis melo
**miR162**	miR162	21	TCGATAAACCTCTGCATCCAG	297	273	179	159	Cucumis melo
**miR164**	miR164b	21	TGGAGAGGCAGGGCACATGCT	12033	11108	9392	8706	Cucumis melo
	miR164d	21	TGGAGAAGCAGGGCACGTGCA	3646	5990	4996	4732	Cucumis melo
**miR166**	miR166a	21	TCGGACCAGGCTTCATTCCCC	612064	812878	688673	731700	Cucumis melo
	miR166e	21	TCTCGGACCAGGCTTCATTCC	47206	65040	49918	62368	Cucumis melo
	miR166f	21	GGAATGTTGTCTGGCTCGAGG	3617	4526	6114	5734	Cucumis melo
	miR166g	21	TCGGACCAGGCTTCATTCCCG	83581	113871	110973	115851	Cucumis melo
**miR167**	miR167b	21	TGAAGCTGCCAGCATGATCTA	28424	26010	19104	19164	Cucumis melo
	miR167c	21	TGAAGCTGCCAGCATGATCTT	130745	107271	116140	99927	Cucumis melo
	miR167f	22	TGAAGCTGCCAGCATGATCTGA	231234	232791	63348	50813	Cucumis melo
**miR168**	miR168b	21	TCGCTTGGTGCAGGTCGGGAA	125549	143512	101662	136051	Malus domestica
**miR169**	miR169g	21	AAGCCAAGGATGAATTGCCGG	7931	13434	6088	12336	Cucumis melo
**miR171**	miR171c	21	GATGTTGGAACGGTTCAATCA	430	1733	1884	7890	Cucumis melo
**miR172**	miR172c	21	AGAATCTTGATGATGCTGCAT	11217	13842	13372	16093	Cucumis melo
**miR2111**	miR2111b	21	TAATCTGCATCCTGAGGTTTA	291	291	361	518	Cucumis melo
**miR2911**	miR2911	22	GCCGGCCGGGGGACGGACTGGG	41292	91335	23484	25862	Helianthus annuus
	miR2911	24	GCCGGCCGGGGGACGGACTGGGAA	37958	53696	32945	29644	Nicotiana tabacum
**miR2950**	miR2950	21	TTCCATCTCTTGCACACTGGA	510	496	1569	2467	Vitis vinifera
**miR319**	miR319	21	AGTGAATGATGCGGGAGACAA	173	112	369	409	Hevea brasiliensis
**miR390**	miR390c	21	AAGCTCAGGAGGGATAGCGCC	2907	4824	1916	2532	Cucumis melo
**miR396**	miR396b	21	TTCCACAGCTTTCTTGAACTG	2550	2831	2045	2473	Cucumis melo
**miR397**	miR397	21	TCATTGAGTGCAGCGTTGATG	152	224	349	478	Cucumis melo
**miR408**	miR408	21	ATGCACTGCCTCTTCCCTGGC	121	109	342	410	Cucumis melo
**miR477**	miR477a	21	ACCTCCCTCAAAGGCTTCCAA	48	135	352	867	Cucumis melo
**miR530**	miR530b	21	TGCATTTGCACCTACACCTTC	758	657	1220	1473	Cucumis melo
**miR858**	miR858	22	TCTCGTTGTCTGTTCGACCTTG	118	87	125	265	Cucumis melo
**miR894**	miR894	20	GTTTCACGTCGGGTTCACCA	1259	2526	1575	3292	Populus trichocarpa

### Identification of novel miRNAs

In addition to conserved miRNAs, we identified 33 new miRNA candidates in the four libraries. These miRNAs were classified as new pumpkin miRNAs and named as “novel”, and five novel miRNAs, novel_mir_17, novel_mir_19, novel_mir_34, novel_mir_36, and novel_mir_39, were identified in both libraries (S3 Table). Twelve novel miRNAs, such as novel_mir_4, novel_mir_11, and novel_mir_15, were only expressed in *C*. *maxima* Duch., and four novel miRNAs, novel_mir_10, novel_mir_29, novel_mir_31, and novel_mir_33, were uniquely expressed in 24hNR. Ten novel miRNAs, such as novel_mir_96, novel_mir_102, novel_mir_111, and novel_mir_128, were only expressed in *C*. *moschata* Duch., and two novel miRNAs, novel_mir_101, novel_mir_108, were uniquely expressed in 54hNR. The length of the newly identified miRNAs ranged from 20 bp to 23 bp in length, and the minimal folding free energies varied from -109.2 kcal mol ^-1^ to -20 kcal mol ^-1^ according to Mfold. These findings were similar to the free energy values of other plant miRNA precursors. These novel miRNAs showed different abundance levels in the four libraries. Novel_mir_19 and novel_mir_36 were identified in both libraries, but the former contained an average of 48 reads and the latter contained 7,823 reads. Novel_mir_4 and novel_mir_11, which were only expressed in *C*. *maxima* Duch., contained an average of 10 and 427 reads, respectively. Moreover, novel_mir_111 and novel_mir_113, which were uniquely expressed in *C*. *moschata* Duch., comprised an average of 13,393 and 41 reads, respectively.

### Differential expression of miRNAs between *Cucurbita* germplasm

We compared 24hR and 24hNR (24hR-vs-24hNR), as well as 54hR and 54hNR (54hR-vs-54hNR), to identify the differences in expression of conserved and novel miRNAs in the two *Cucurbita* germplasm. 24hR-vs-24hNR and 54hR-vs-54hNR reflected the differences in the responses of two *Cucurbita* germplasm under salt stress conditions. Ten conserved miRNAs and six novel miRNAs were up-regulated in 24hR-vs-24hNR, whereas two conserved miRNAs and four novel miRNAs were down-regulated in 24hR-vs-24hNR as shown in Tables 4 and 5. Meanwhile, 13 conserved miRNAs and six novel miRNAs were up-regulated in 54hR-vs-54hNR, whereas only one novel miRNA was down-regulated in 54hR-vs-54hNR.

**Table 4:** Different expression of conserved miRNAs in the two *Cucurbita* germplasm response to salt stress.

miRNA序列	Family	N12 Log2(salt/ctrl)	N15 Log2(salt/ctrl)	Putative target
TGGAGAAGCAGGGCACGTGT	miR164	1.19	1.40	NAC domain-containing protein
CCCGCCTTGCATCAACTGAAT	miR168	1.66	1.01	Cytoplasmic tRNA 2-thiolation protein 2
AGCCAAGGATGAATTGCCGG	miR169	2.30	2.61	Nuclear transcription factor Y subunit
				Methyl-CpG-binding domain-containing protein
AGCTCAGGAGGGATAGCGCC	miR390	1.60	1.13	Dehydration-induced protein
ACCTCCCTCAAAGGCTTCCAA	miR477	1.01	1.01	
GTTTCACGTCGGGTTCACCA	miR894	1.46	1.13	
TGACAGAAGAGAGGGAGCAC	miR156	1.11		Squamosa promoter-binding-like protein
GCTCTCTATGCTTCTGTCATCA	miR157	2.77		
TCTCGGATCAGGCTTCATTCC	miR166	1.68		Respiratory burst oxidase homolog protein
TGGGAATCTTGATGATGCTGC	miR172	1.30		Ethylene-responsive transcription factor
AGGCATCGGGGGCGCAACGC	miR1310	-1.69		Methyltransferase
TGCCTGGCTCCCTGTATGCCA	miR160	-1.14		Auxin response factor
AGGGGAATGTTGTCTGGCTCG	miR166		1.53	glycerol-3-phosphate acyltransferase
TGATTGAGCCGTGCCAATATC	miR171		1.04	Cell wall structural protein
GCAGCACCATCAAGATTCACA	miR172		2.12	Gibberellin-regulated protein 14
				Phytochrome C
TTGGACTGAAGGGAGCTCC	miR319		2.29	TCP family transcription factor
				Zinc finger A20 and AN1 domain-containing stress-associated protein
GTTCAATAAAGCTGTGGGAA	miR396		1.39	Zinc finger domain-containing protein
				Eukaryotic translation initiation factor 4G
AACCTGGCTCTGATACCA	miR5139		1.21	F-box/WD-40 repeat-containing protein
CCGACCTTAGCTCAGTTGG	miR6478		1.40	

**Table 5:** Different expression of novel miRNAs in the two *Cucurbita* germplasm response to salt stress.

miRNA序列	Family	N12 Log2(salt/ctrl)	N15 Log2(salt/ctrl)	Putative target
GTTCATGAAAGCTGTGGGAGA	novel_mir_42	12.31	12.29	Protein phosphatase 2C
TGGAGAAGCAGGGCACGTGCTG	novel_mir_74	-8.46	9.37	NAC domain-containing protein
GGCGGAACGCGCACGGTGGGGCA	novel_mir_10	11.17		
TGTAGATGATCGTTCTCGGGT	novel_mir_21	9.69		
AGGGATCAAAGAGAGACAGAGA	novel_mir_25	8.02		Splicing factor U2af large subunit B
TCTCTAGTAGACGTATTTTA	novel_mir_29	6.70		bHLH family transcription factor
				Cation/H(+) antiporter 18
AGATCATGCGACAGTTTCACC	novel_mir_31	8.24		
AAGCTGTGATGAAAATCTTCT	novel_mir_15	-1.96		WRKY family transcription factor
				Ethylene-responsive transcription factor
				MYB family transcription factor
				bHLH family transcription factor
				Trihelix transcription factor GT-2
				Auxin-induced in root cultures protein
				Proline-rich receptor-like protein kinase PERK2
				G-box-binding factor 4
				Monothiol glutaredoxin-S7
				RING-H2 finger protein
				Hypersensitive-induced response protein 3
				Growth-regulating factor
AAGGAATGAAGGAAGCATGG	novel_mir_79	-10.30		Calcium-dependent protein kinase
TTTAAAACGCGTCTACTAGGG	novel_mir_80	-7.32		CBL-interacting serine/threonine-protein kinase 3
				Nitrate reductase
				Peroxidase 47
				Receptor-like protein kinase 5
				1-aminocyclopropane-1-carboxylate oxidase homolog 1
				Glycerate dehydrogenase
				Mitogen-activated protein kinase
GCGTCTGTTAGGGAGAGGTTTCC	novel_mir_101		7.63	CBL-interacting serine/threonine-protein kinase
				1-aminocyclopropane-1-carboxylate oxidase homolog 1
				Proline-rich receptor-like protein kinase
				Transcription factor ILR3
				Zinc finger protein CONSTANS-LIKE 2
				LRR receptor-like serine/threonine-protein kinase
TCACCGCTAGTAGATATTGTT	novel_mir_108		7.21	ABA-INSENSITIVE 5-like protein
				Auxin efflux carrier component 1
CGAAAATTAAGCCTACAATCA	novel_mir_113		1.17	
CTTAGCCAACGACACGTGCCT	novel_mir_128		1.36	
TGCGATTGTGACAAGTGGTATC	novel_mir_142		-10.64	Early nodulin

We also compared 24hR-vs-24hNR and 54hR-vs-54hNR to determine the differences in the response of the two *Cucurbita* germplasm to salt stress based on the deduced background. Seven miRNAs (six conserved miRNAs and one novel miRNAs) were both up-regulated in 24hR-vs-24hNR and 54hR-vs-54hNR, whereas only novel_mir_74 was down-regulated in 24hR-vs-24hNR but up-regulated in 54hR-vs-54hNR. Nine miRNAs (four conserved miRNAs and five novel miRNAs) were up-regulated in 24hR-vs-24hNR, whereas 12 miRNAs (seven conserved miRNAs and five novel miRNAs) were up-regulated in 54hR-vs-54hNR. Moreover, five miRNAs (two conserved miRNAs and three novel miRNAs) were down-regulated in 24hR-vs-24hNR, whereas just novel_mir_142 was down-regulated in 54hR-vs-54hNR. In the seven up-regulated miRNAs of the two varieties, novel_mir_42 (12.31-fold in 24hR-vs-24hNR and 12.29-fold in 54hR-vs-54hNR) highly increased, whereas miRNA164, miRNA168, miRNA169, miRNA390, and miRNA477 only slightly increased. Partial novel miRNAs (up- or down-regulated in 24hR-vs-24hNR and 54hR-vs-54hNR) exhibited a higher increase than the conserved miRNAs (Tables 4 and 5). These results indicate that novel miRNAs might have an important role in the salt stress responses of the two *Cucurbita* germplasm.

### Identification of target genes of differentially expressed miRNAs

Differentially expressed conserved and novel miRNAs in 24hR-vs-24hNR and 54hR-vs-54hNR were obtained using the PsRNAtarget method to predict putative target genes. The transcriptome sequences of *C*. *maxima* Duch. and *C*. *moschata* Duch. were used as the reference set.

The target genes of majority of conserved miRNAs were determined and conserved across several plant species. Most of these target genes were classified as transcription factors, such as NAC domain-containing protein (miR164), nuclear transcription factor Y subunit (miR169), ethylene-responsive transcription factor (miR172), auxin response factor (miR160), and TCP family transcription factor (miR319). Some of these genes were also categorized as functional proteins, such as dehydration-induced protein (miR390), glycerol-3-phosphate acyltransferase (miR166), cell wall structural protein (miR171), gibberellin-regulated protein 14 (miR172), and phytochrome C (miR172), all of which were involved in plant metabolism and environmental stimulus responses (Table 4).

The target genes of a small portion of novel miRNAs were successfully predicted. A total of 287 target genes were recognized for seven novel miRNAs (S4 Table) with an average of 41 in 24hR-vs-24hNR, whereas 152 target genes were recognized for six novel miRNAs with an average of 25 in 54hR-vs-54hNR.

Most target genes of novel miRNAs were also classified as transcription factors, such as NAC domain-containing protein, WRKY family transcription factor, ethylene-responsive transcription factor, MYB family transcription factor, bHLH family transcription factor, trihelix transcription factor GT-2, and RING-H2 finger protein. Moreover, some target genes, including proline-rich receptor-like protein kinase, calcium-dependent protein kinase, nitrate reductase, and LRR receptor-like serine/threonine-protein kinase, were involved in diverse biological processes. In addition, partial target genes, such as cation/H^+^ antiporter 18, CBL-interacting serine/threonine-protein kinase, auxin-induced in root cultures protein, monothiol glutaredoxin-S7, hypersensitive-induced response protein 3, peroxidase 47, and 1-aminocyclopropane-1-carboxylate oxidase homolog 1, were involved in responses to salt treatment. These results further demonstrated that novel miRNAs participated in the responses of the two *Cucurbita* germplasm to salt treatment.

### GO enrichment analysis of target genes of conserved miRNAs in two *Cucurbita* germplasm

GO is an international standardized classification system for gene function. A set of controlled vocabulary is used to comprehensively describe the property of genes and gene products.

**Fig 4:**
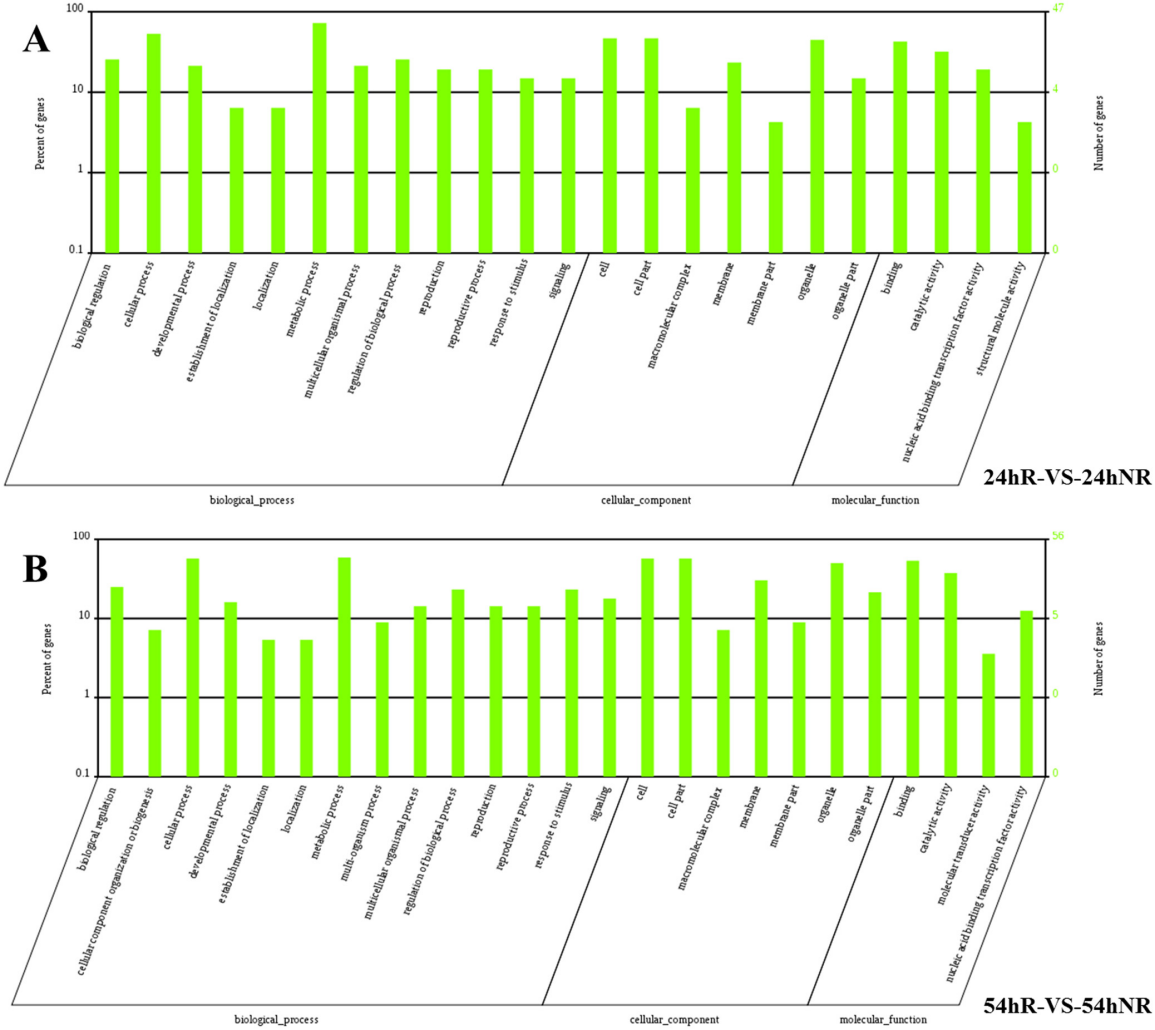
Gene Ontology classifications of the target genes of conserved miRNAs in the two *Cucurbita* germplasm.

To clearly understand the regulatory roles of miRNAs in salt stress, we performed an analysis of GO-based term classification on target genes of the differentially expressed conserved miRNAs in 24hR-vs-24hNR and 54hR-vs-54hNR. In total, 387 target genes were categorized to 12 biological processes, seven molecular functions, and four cellular components in 24hR-vs-24hNR, whereas 546 target genes were categorized to 14 biological processes, seven molecular functions and four cellular components in 54hR-vs-54hNR. The top five biological processes in 24hR-vs-24hNR were cellular process, metabolic process, biological regulation, regulation of biological process and developmental process (Fig 4A). In 54hR-vs-54hNR, there were the same order in top four biological processes followed by response to stimulus and signaling (Fig 4B). The main cellular components in 24hR-vs-24hNR and 54hR-vs-54hNR were cell, cell part, and organelle. Regarding molecular functions, all genes were clustered into binding, catalytic activity, nucleic acid binding transcription factor activity and structural molecule activity in 24hR-vs-24hNR and 54hR-vs-54hNR.

### Confirmation of predicted miRNAs and target genes by qRT-PCR

**Fig 5:**
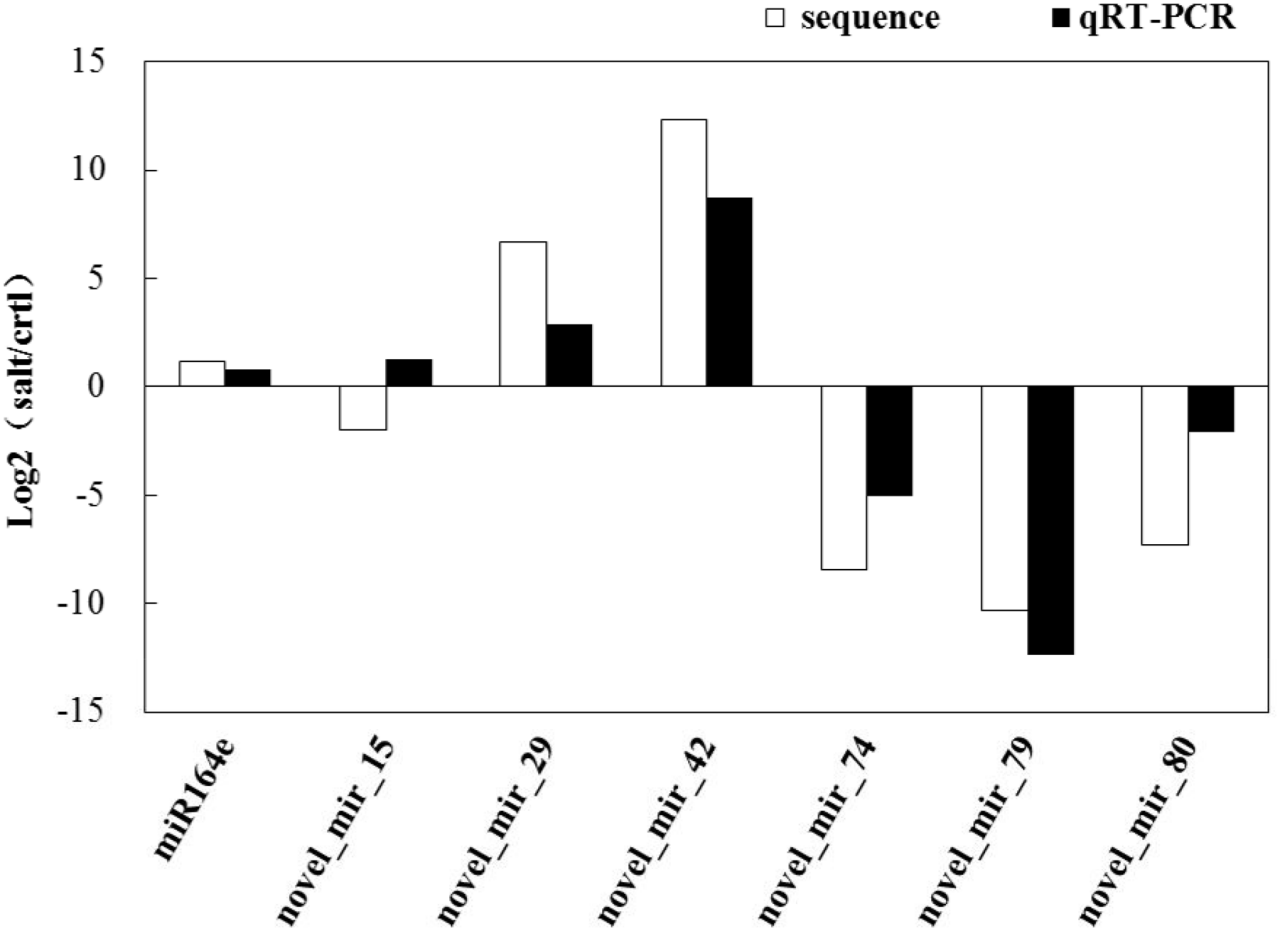
Expression analysis of miRNAs in N12 by qRT-PCR. Column above the X-axis indicates up-regulated miRNAs; column below the X-axis represents down-regulated miRNAs.

**Fig 6:**
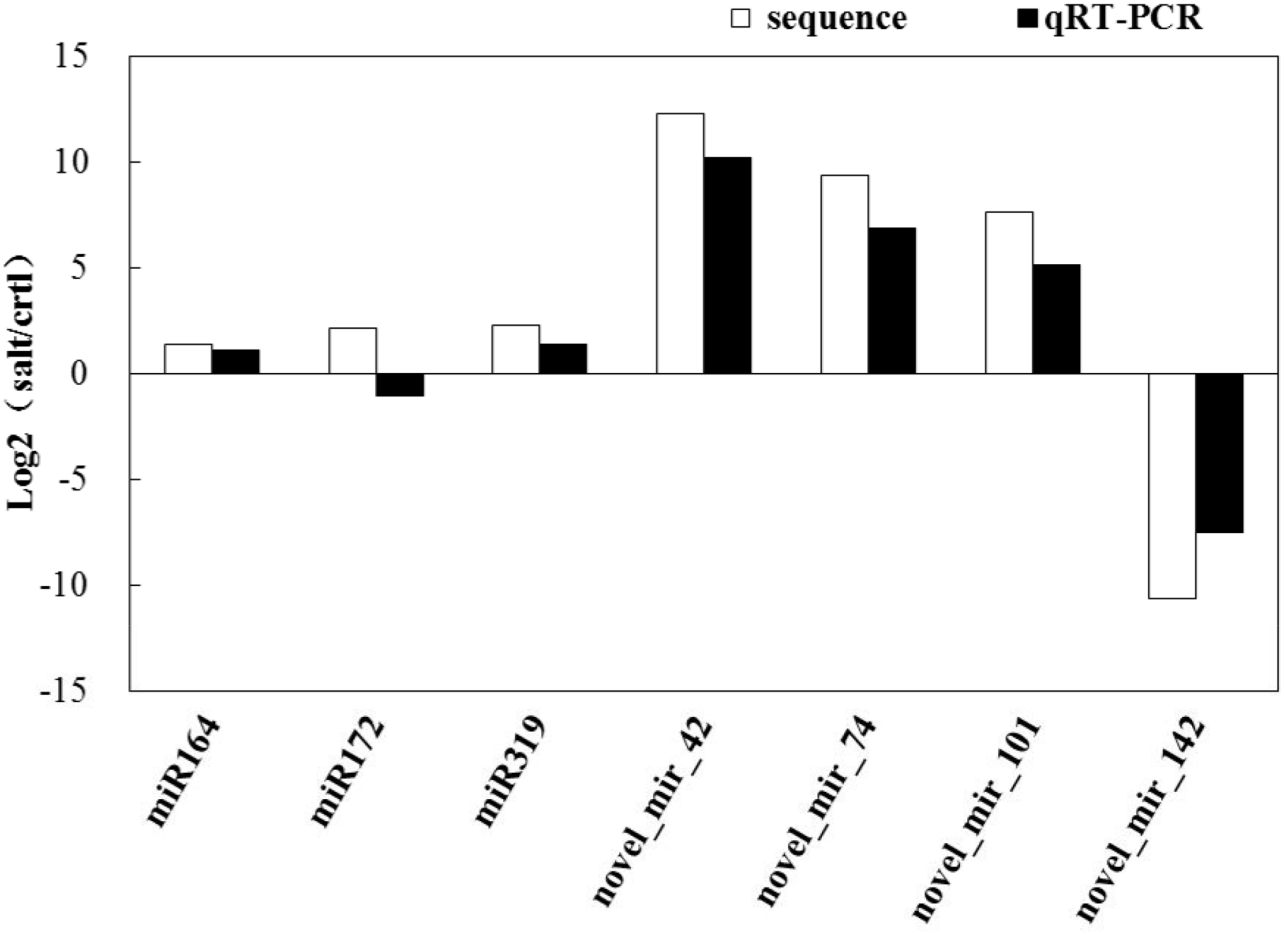
Expression analysis of miRNAs in N15 by qRT-PCR. Column above the X-axis indicates up-regulated miRNAs; column below the X-axis represents down-regulated miRNAs.

We selected several miRNAs with different expression patterns in N12 and N15 for qRT-PCR analysis. The expression patterns of the most miRNAs obtained by qRT-PCR were consistent with the results of Illumina sequencing (Figs 5 and 6).

**Fig 7:**
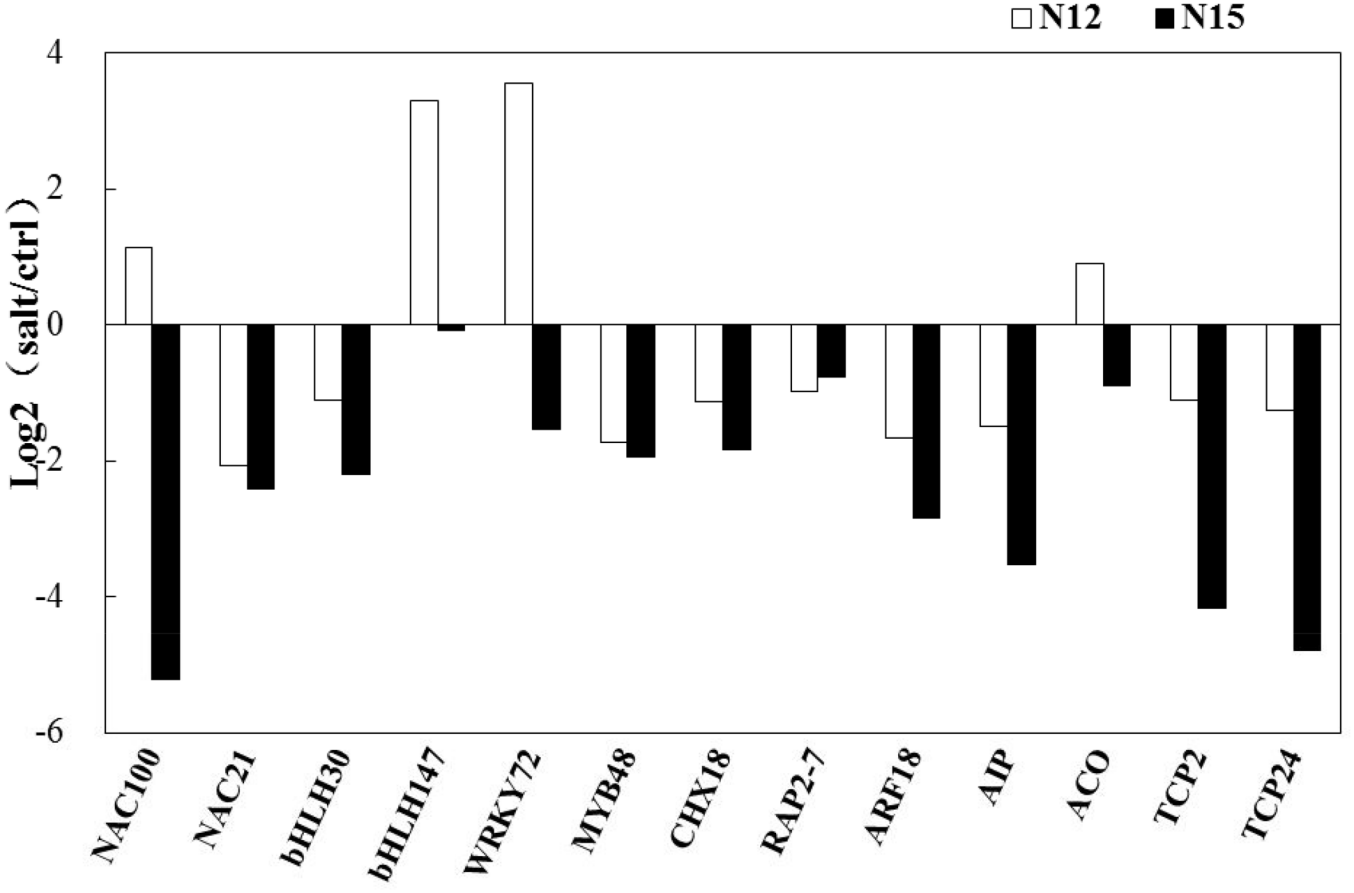
Expression analysis of the targets by qRT-PCR in N12 and N15. Column above the X-axis indicates up-regulated mRNAs; column below the X-axis represents down-regulated mRNAs.

Moreover, several target genes of miRNAs were transcription factors or involved in responses to salt treatment. These target genes were also selected for qRT-PCR analysis. Most of these genes were clearly down-regulated after salt treatment in the two *Cucurbita* germplasm, but some transcription factors (NAC100, bHLH147, and WRKY72) were only up-regulated in N12 (Fig 7).

## Discussion

High-throughput sequencing is used to systematically identify plant miRNA responses to abiotic stresses. It has greatly enriched knowledge on miRNAs under adverse environmental conditions. As a major environmental stress factor, salinity reduces the growth and productivity of cucurbits. However, grafting onto pumpkin rootstock can increase salt tolerance and improve fruit yield of cucurbits by limiting the transport of Na^+^ to shoots [20, 38, 39]. The two *Cucurbita* germplasm, namely, N12 (*C*. *maxima* Duch.) and N15 (*C*. *moschata* Duch.), were used as rootstock and demonstrated different Na^+^ accumulation patterns. N12 accumulated Na^+^ in the shoots, whereas N15 accumulated Na^+^ in the roots. Scanning ion-selective electrode technique demonstrated that the roots of a salt-tolerant pumpkin cultivar (Chaojiquanwang, *C*. *moschata* Duch.,) exhibited high capacity to exclude Na^+^ [40]. This finding explained that less Na^+^ was transported from the roots to the shoots in N15.

### Selection of the time point and plant tissue for small RNA sequencing

Long term stress revealed the different expression levels of miRNAs and consequences of the stressed plants. The mechanisms by which plants adapt to abiotic stresses should be further investigated with regard to their gene expression under short-term stress (less than4 h) conditions when plants can survive the stress [41]. To obtain sufficient information about the response of the two *Cucurbita* germplasm to salt stress in the miRNA level, we select short-term salt stress samples N12 and N15 for small RNA sequencing.

Plant roots are the primary site of perception. There are highly sensitive organs to salt stress, and transmit the salt signal to shoots for proper salt response and adaptation of the plant. Therefore, elucidating the metabolic mechanisms and signaling of salt responses in roots is critical to improve plant salt tolerance [42, 43]. Root tissues under short-term salt stress of N12 and N15 were used to determine small RNA populations in our study.

### Overview of the sequencing data

To explore the regulatory mechanism of miRNAs in different Na^+^ accumulation patterns of the pumpkins under short-term salt stress, we identified and characterized the miRNAs from the root tissues of the two *Cucurbita* germplasm, namely, N12 (*C*. *maxima* Duch.) and N15 (*C*. *moschata* Duch.) under salt treatment and control after 4 h. The present study at first time investigated miRNAs in different Na^+^ accumulation patterns in relation to the responses of pumpkins to salt stress. The results provide new information on novel miRNAs and target genes in *cucurbita* root tissues.

More than 20,000,000 reads of 18–30 nt were obtained from the four libraries (24hR, 24hNR, 54hR, and 54hNR). A total of 1529, 1643, 1561, and 1820 conserved miRNAs were identified in the 24hR, 24hNR, 54hR and 54hNR libraries, respectively. A few highly conserved miRNA families, including MiR156 and MiR167, exhibited high numbers of reads in the four libraries, whereas other families, such as miR398, showed very low abundance. Our result were supported by several studies and demonstrated that these miRNAs respond to salt stress [10, 11].

### Identification of conserved miRNAs and target gene

In long evolutionary timescales, the regulatory interactions of well-conserved miRNA families contradict the findings with regard to their involvement in development; hence, these units of post-transcriptional gene control are indispensable during diversification of land plants [44]. Remarkably, most conserved miRNAs are significantly altered in response to salinity. The up-regulation of miR168 and miR169 during salt stress has been commonly observed in Arabidopsis, rice, maize, and grapevine [3, 12, 45]. Most conserved miRNAs, such as miR164, miR168, miR169, and miR390 were up-regulated in the two *Cucurbita* germplasm with targets genes as diverse transcription factors, such as NAC domain-containing protein, nuclear transcription factor Y subunit, and dehydration-induced protein (Table 4). These results demonstrated that the two *Cucurbita germplasm* share universal mechanism of salt-stress responses.

In addition to the well-conserved miRNAs that were up-regulated in the two *Cucurbita* germplasm, some up- or down-regulated miRNAs in N12 or N15, such as miR156, miR160, miR172, and miR319, interacted with squamosa promoter-binding-like protein, auxin response factor, gibberellin-regulated protein 14, and TCP family transcription factor. These miRNAs seems to play important roles in variety-specific pumpkin growth and development.

### Novel miRNAs play critical roles in the response to salt treatment

Given that miRNAs are complementary to miRNA* sequences in the precursor, miRNA* sequences of novel miRNA were also needed to be found in sequencing data, and other criteria established by miRBase were also needed to be met [46]. According to these criteria, a total of 33 new miRNA candidates were identified in the four libraries. The target genes of novel miRNAs were also enriched in transcription factors responses to salt stress. However, most novel miRNAs showed different expression levels in the two *Cucurbita* germplasm. One of the novel miRNAs (novel_mir_74) was down-regulated in 24hR-vs-24hNR but up-regulated in 54hR-vs-54hNR, and its target gene was NAC domain-containing protein. The target genes of novel_mir_80 and novel_mir_101 were CBL-interacting serine/threonine-protein kinase and 1-aminocyclopropane-1-carboxylate oxidase homolog 1, respectively. The former novel miRNA was down-regulated in 24hR-vs-24hNR, whereas the latter was up-regulated in 54hR-vs-54hNR. NAC domain-containing protein functions as a regulator in various stress signaling pathways [47]. CBL-interacting serine/threonine-protein kinase is a member of the SOS salt-tolerant pathway [6]. 1-Aminocyclopropane-1-carboxylate oxidase homolog 1 is the key enzyme that controls and regulates ethylene production in plants, and it is up-regulated by various abiotic and biotic stresses [48]. These findings indicated that the SOS salt-tolerant pathway and ethylene may play an important role in N12 response to salt stress, whereas NAC domain-containing protein was more critical to N15 response to salt stress. Moreover, novel_mir_79 and novel_mir_80 were evidently down-regulated in 24hR-vs-24hNR; the target genes of these miRNAs were protein kinases, such as calcium-dependent protein kinase, receptor-like protein kinase 5, and mitogen-activated protein kinase. These protein kinases function as the key regulators of salt stress and ABA signaling in plants [43].

In the present study, novel miRNAs were highly increased or decreased after salt stress, and most target genes were transcription factors and salt stress-responsive proteins. Therefore, novel miRNAs may play critical roles in the response of the two *Cucurbita* germplasm to salt treatment.

## Conclusion

Two *Cucurbita* germplasm, namely, N12 (*C*. *maxima* Duch.) and N15 (*C*. *moschata* Duch.), exhibited different Na^+^ accumulation patterns. N12 accumulated Na^+^ in the shoots, whereas N15 accumulated Na^+^ in the roots. High-throughput Illumina sequencing showed that the two *Cucurbita* germplasm contained 58 conserved miRNA families and 33 novel miRNAs. The differential expression of conserved miRNAs and novel miRNAs under salt stress conditions between the two varieties and their target genes indicated that novel miRNAs might play important roles in the response of the two *Cucurbita* germplasm to salt treatment. The present study at first time investigated small RNAs in the response of pumpkin to salt stress. The results provide new information regarding novel miRNAs and their target genes in *cucurbita*, and will contribute to the breeding of *Cucurbita* rootstocks.

## Supporting Information

S1 File First nucleotide bias of novel miRNA in the four libraries. 24hR, N12 root under control library; 24hNR, N12 root under salt stress treatment library; 54hR, N15 root under control library; 54hNR, N15 root under salt stress treatment library.
(PDF)

S2 File Secondary structures of identified novel miRNAs. (PDF)

S1 Table Primer sequences used for qRT-PCR. (XLSX)

S2 Table Conserved miRNAs in the four libraries. 24hR, N12 root under control library; 24hNR, N12 root under salt stress treatment library; 54hR, N15 root under control library; 54hNR, N15 root under salt stress treatment library.
(XLSX)

S3 Table Novel miRNAs and their expression levels among the four libraries. 24hR, N12 root under control library; 24hNR, N12 root under salt stress treatment library; 54hR, N15 root under control library; 54hNR, N15 root under salt stress treatment library.
(XLSX)

S4 Table Identified target genes of differentially expressed conserved and novel miRNAs in 24hR-vs-24hNR. 24hR, N12 root under control library; 24hNR, N12 root under salt stress treatment library; 54hR, N15 root under control library; 54hNR, N15 root under salt stress treatment library.
(XLSX)

S5 Table Identified target genes of differentially expressed conserved and novel miRNAs in 54hR-vs-54hNR. 24hR, N12 root under control library; 24hNR, N12 root under salt stress treatment library; 54hR, N15 root under control library; 54hNR, N15 root under salt stress treatment library.
(XLSX)

S6 Table GO enrichment results with description of GO accessions and corresponding genes in 24hR-vs-24hNR. 24hR, N12 root under control library; 24hNR, N12 root under salt stress treatment library; 54hR, N15 root under control library; 54hNR, N15 root under salt stress treatment library.
(XLSX)

S7 Table GO enrichment results with description of GO accessions and corresponding genes in 54hR-vs-54hNR. 24hR, N12 root under control library; 24hNR, N12 root under salt stress treatment library; 54hR, N15 root under control library; 54hNR, N15 root under salt stress treatment library.
(XLSX)
