# Supplementary material for: High Throughput Sequencing of Small RNAs in the Two Cucurbita Germplasm with Different Sodium Accumulation Patterns Identifies Novel MicroRNAs Involved in Salt Stress Response
Source: PLoS One. 2015 May 26;10(5):e0127412. doi: 10.1371/journal.pone.0127412 (PMC4444200; doi:10.1371/journal.pone.0127412)

S1 File First nucleotide bias of novel miRNA candidates in the four libraries.

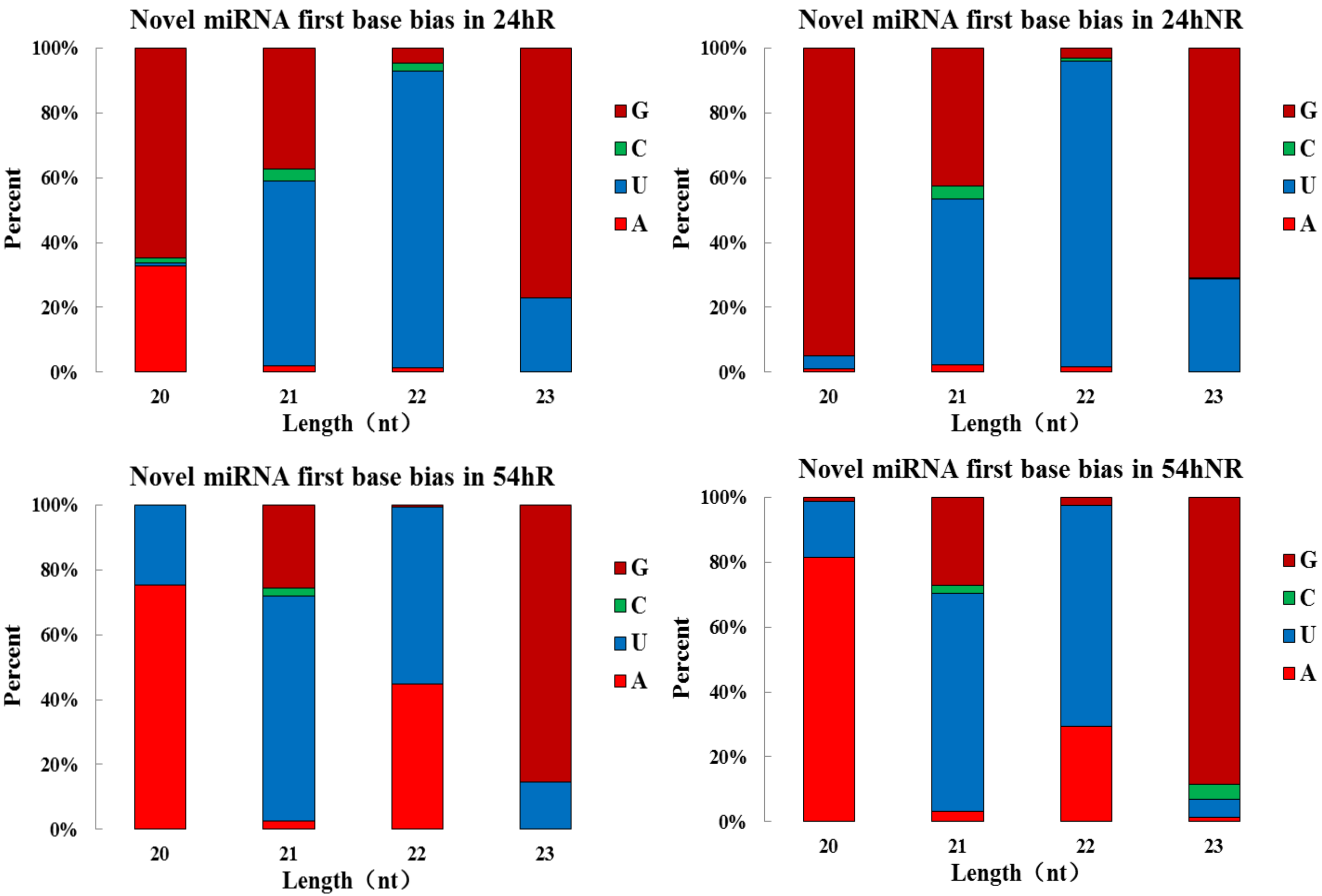

Supplement: S1 File — 24hR, N12 root under control library; 24hNR, N12 root under salt stress treatment library; 54hR, N15 root under control library; 54hNR, N15 root under salt stress treatment library. (PDF) [file pone.0127412.s001.pdf]
