# Supplementary material for: High Throughput Sequencing of Small RNAs in the Two Cucurbita Germplasm with Different Sodium Accumulation Patterns Identifies Novel MicroRNAs Involved in Salt Stress Response
Source: PLoS One. 2015 May 26;10(5):e0127412. doi: 10.1371/journal.pone.0127412 (PMC4444200; doi:10.1371/journal.pone.0127412)
Supplement: S2 File — (PDF) [file pone.0127412.s002.pdf]

S2 File Secondary structures of identified novel miRNAs.

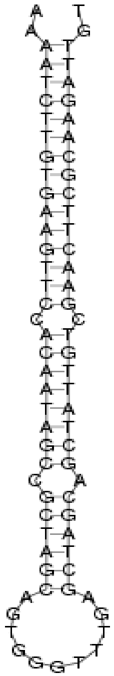

novel-mir-4

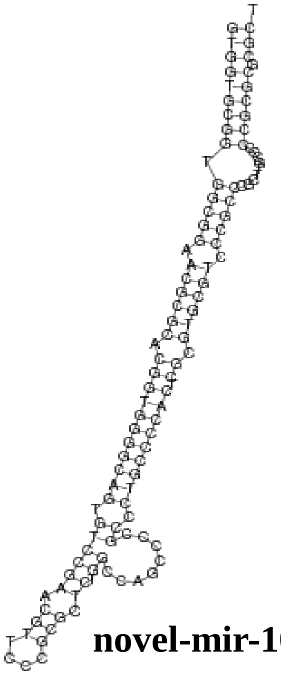

novel-mir-10

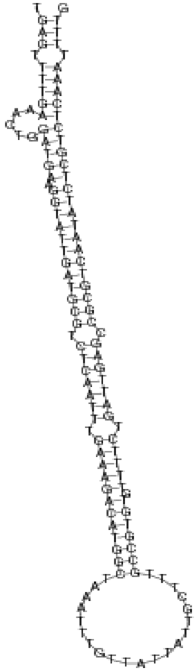

novel-mir-11

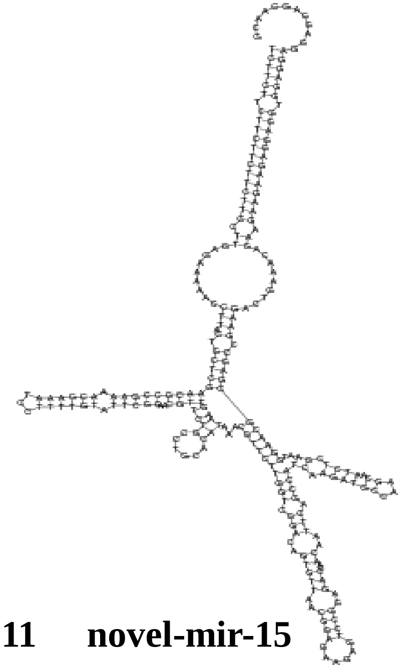

novel-mir-15

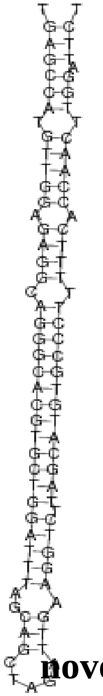

novel-mir-17

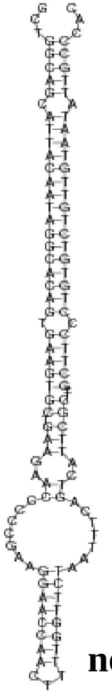

novel-mir-19

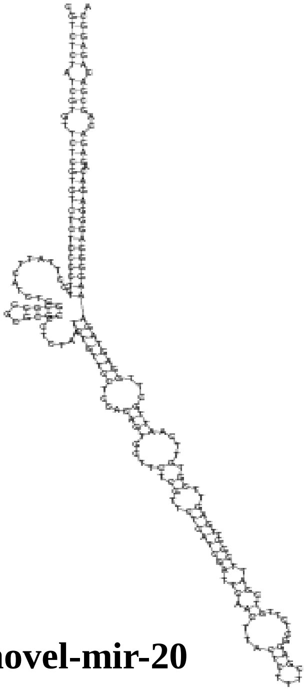

novel-mir-20

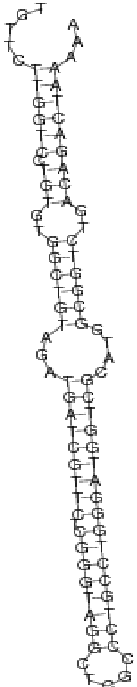

novel-mir-21

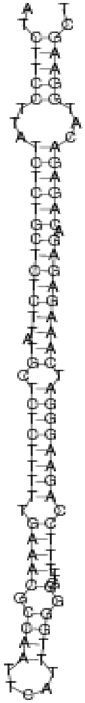

novel-mir-25

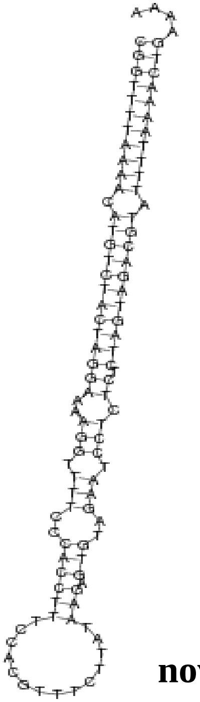

novel-mir-29

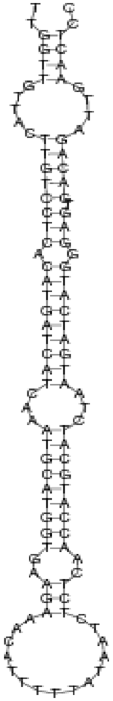

novel-mir-30

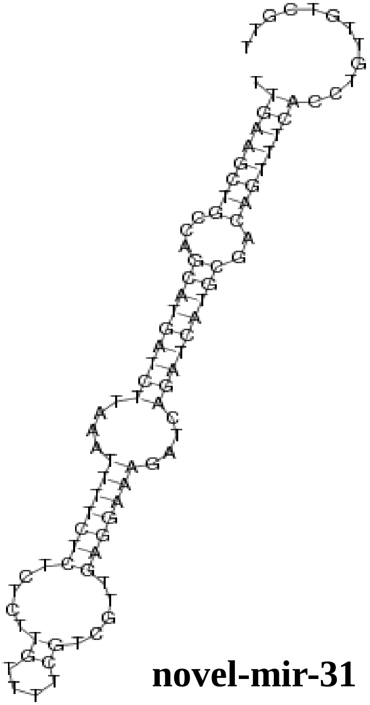

novel-mir-31

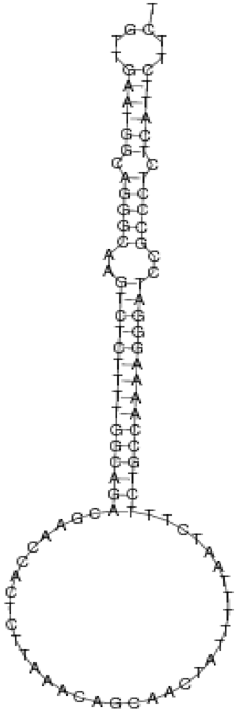

novel-mir-33

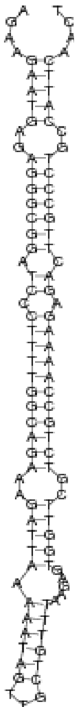

novel-mir-34

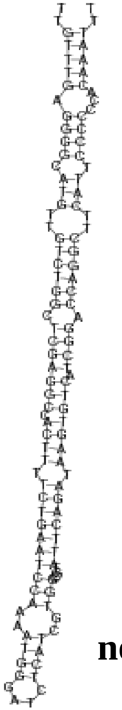

novel-mir-36

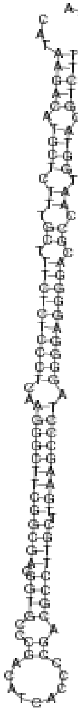

novel-mir-39

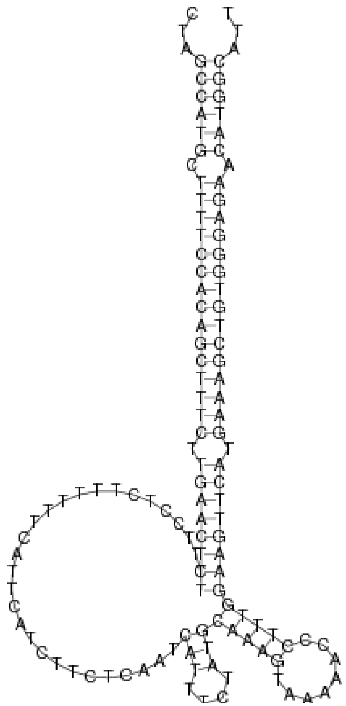

novel-mir-42

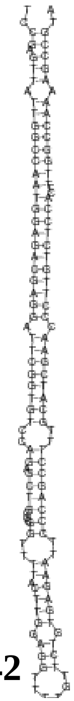

novel-mir-45

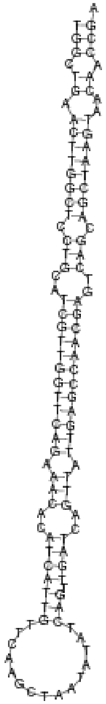

novel-mir-61

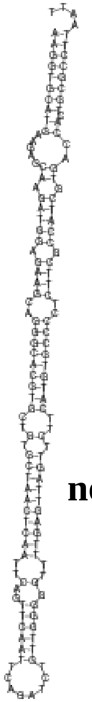

novel-mir-74

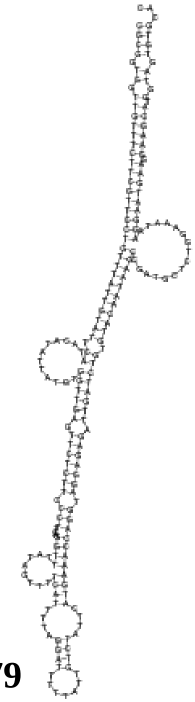

novel-mir-79

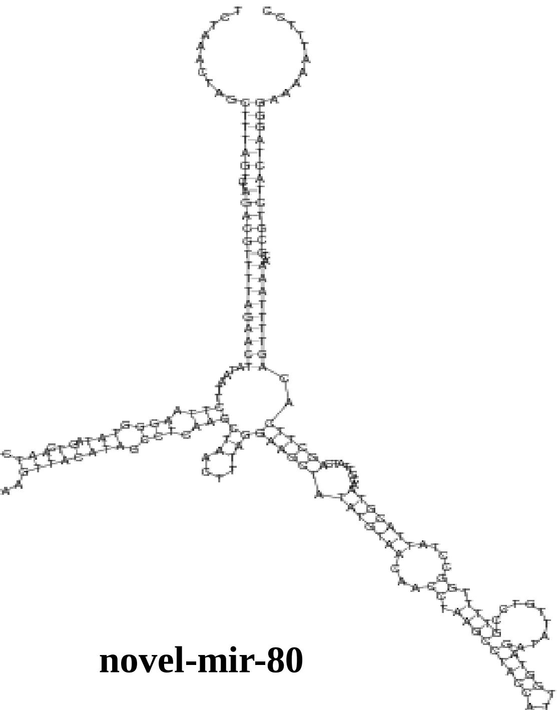

novel-mir-80

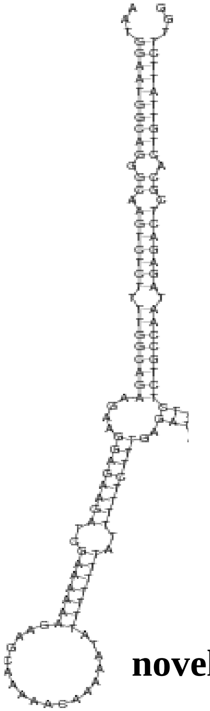

novel-mir-84

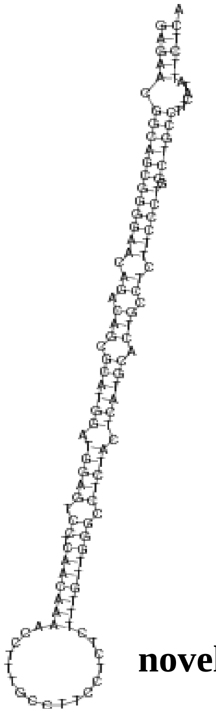

novel-mir-96

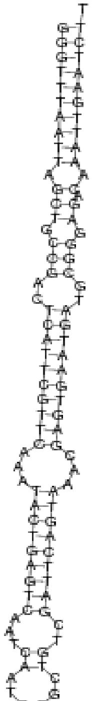

novel-mir-100

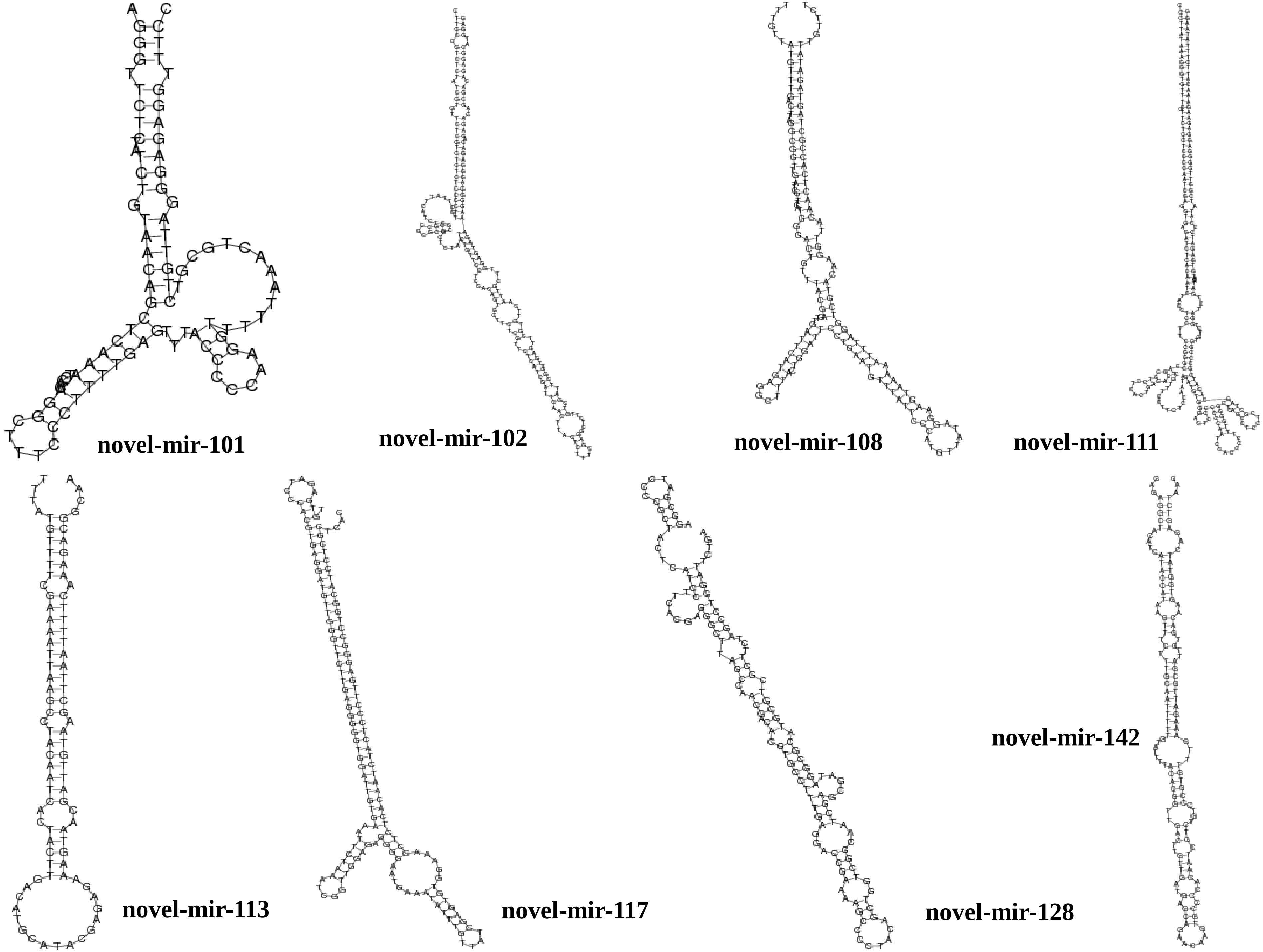
novel-mir-101
novel-mir-102
novel-mir-108
novel-mir-111
novel-mir-113
novel-mir-117
novel-mir-128
novel-mir-142
